# Language Did Not Spring Forth 100,000 Years Ago

**DOI:** 10.1371/journal.pbio.1002064

**Published:** 2015-02-13

**Authors:** Philip Lieberman

**Affiliations:** Cognitive, Linguistic and Psychological Sciences, Brown University, Providence, Rhode Island, United States of America

## Abstract

In response to an Essay by Johan Bolhuis and co-authors, Phillip Lieberman contends that rather than arising from a key recent innovation ("merge"), language arose by gradual evolution of ancient capabilities.

Language evolved over millions of years by Darwinian processes, and its primary role is communication. Speech is the default mode by which we share our thoughts with others. The communicative role of language is apparent in that the neural structures that code a word’s meaning in the brain are activated by the sound pattern of its name [1]. In their essay [2], Bolhuis et al. [2] argue that speech and communication are irrelevant and instead describe a “language faculty” that consists of a single operation, “*merge*,” which is argued to have suddenly come into being 70 to 100 thousand years ago. Bolhuis et al. [2] and Chomsky [3] therefore argue against natural selection playing a role in the evolution of language.

“Recursive” syntax supposedly is the defining characteristic of language. Noam Chomsky has claimed that children do not learn their native languages through imitation and associative learning. He instead proposed an innate “Universal Grammar” (UG) [4] that specified numerous “principles and parameters” [5] that when activated determined a language’s syntax. UG, however, is implausible owing to genetic variation. In some children, the principles and parameters necessary to acquire their native language would be missing, though other languages could be acquired [6,7]. The “Strong Minimalist Thesis” described in Bolhuis et al. [2] instead claims that a single innately transmitted operation, *merge*, which yields complex sentences with embedded clauses, accounts for the acquisition of language. *Merge* “takes exactly two (syntactic) elements […] and puts them together” [2]. But *merge* entails having the same comprehensive innate knowledge base as UG because different languages put different things together. A child exposed to English will hear two-word, definite-article noun sequences such as *the cow*. However, Chinese does not use definite articles. If languages were not learned using the cognitive processes by which we learn how to use chopsticks or forks, the article-noun constructions and thousands of language-specific aspects of syntax would have had to have been innately specified 100,000 years ago to account for the diversity that marks human languages. Both UG and the store of knowledge necessary for *merge* to generate the syntax of every language that existed, exists, or may come into being are implausible. Moreover, selectional constraints, such as those involved in walking and other motor acts, yield hierarchical action patterns, e. g., those underlying dancing, that potentially can generate an infinite number of combinations—in the example here, dances. This argues against the uniqueness and language specificity of the *merge* operation.

Chimpanzees provide some insights on the cognitive and linguistic capabilities of early hominims and point to an initial stage that had words and simple syntax. Enhanced human capabilities appear to derive from mutations and selective sweeps over the past 400 or 500 thousand years that yielded a neural substrate that also regulates cognition, motor control, and other aspects of behavior [6–8]. Data from hundreds of independent studies, ranging from the deficits of aphasia and Parkinson disease to functional magnetic resonance imaging (fMRI) activation patterns, argue against a “faculty of language” committed to language and language alone. Darwin’s “problem” doesn’t exist. He noted “the highly important fact that an organ originally constructed for one purpose…may be converted into one for a wholly different purpose” [9]. Neural circuits linking regions of the cortex with the subcortical basal ganglia and other neural structures regulate motor control (including speech), comprehension of syntax, and a range of cognitive tasks [6–8,10–12]. In these human neural circuits, the basal ganglia, which initially were adapted for motor control, also regulate cognitive and linguistic tasks [6–8,10–12].

One of the central findings of neuroscience is the role of synaptic connectivity and malleability in coding information, transmitting information, and associative learning. Anomalies in the FOXP2 transcriptional factor in humans yield a syndrome—deficits in speech motor control, syntax, and cognition. Over the past 500,000 years, a series of mutations occurred in FOXP2, the genes FOXP2 acts on, and the processes by which they are transcribed. Successive selective sweeps occurred that spread these changes throughout hominin populations. The version of the FOXP2 transcriptional factor—shared by humans, Neanderthals, and Denisovans [13,14]—enhanced synaptic connectivity and malleability in these neural circuits. A later selective sweep on species-specific human genes and mutations affecting the expression of FOXP2 occurred sometime after 260,000 years ago [15,16]. These last genetic events most likely enhanced human cognitive and linguistic capabilities compared to Neanderthals, but they were the end point of a process extending over 500,000 years. When apes are raised in a setting in which language is used, they can acquire and use about 150 words to signal their wants and reflect on past events and assign new, metaphorical referents to these words. The Gardner chimpanzees used American Sign Language (ASL) and mastered elements of its inflectional morphology [17]. The bonobo Kanzi comprehended distinctions in meaning conveyed solely by syntax [18]. Any aspect of language that apes can master most likely was present in the common ancestor of humans and apes and surely was present in Neanderthals, ruling out a “protolanguage” that lacked syntax.

Human speech is a key attribute of language because it allows information to be transmitted at a rate that exceeds the fusion frequency of the auditory system. It otherwise would not be able to retain more than a few words in working memory—precluding comprehending distinctions in meaning conveyed by even moderately complex syntax [19]. The equal proportions of the horizontal and vertical portions of the adult-like human vocal tract allow humans to produce speech sounds that are inherently less susceptible to articulatory errors and less subject to misidentification [20]. These sounds also play a role in the perceptual process that “recovers” the words of a sentence from the acoustic signal [6–8]. However, speech communication is possible with a nonhuman vocal tract, albeit with somewhat higher error rates [21]. The evolution of the human vocal tract thus is consistent with Darwin’s claim for slight advantages steering the course of evolution.

The evolution of the human vocal tract also provides evidence on when hominim brains had linguistic and cognitive capabilities. The low position of the human vocal tract increases the risk of choking on food, which remains the fourth leading cause of accidental death in the United States [22]. The mutations that shaped the human vocal tract would not have been retained unless the neural circuits that allow humans to learn and execute the complex motor acts involved in talking were in place [23]. This capability and the concomitant linguistic and cognitive capabilities of the neural circuits noted above therefore predated the appearance of anatomically modern *Homo sapiens*. As Edmund Crelin and I noted in 1971, Neanderthals must have possessed speech and language, though their vocal tracts precluded their mastering any human dialect [24]. Other evidence argues against Neanderthals lacking language. Neanderthals survived for hundreds of thousands of years in hostile environments, making and wearing clothing, using fire, and burying their dead with grave goods and, as some indications (inscribed rocks and pigments) show, possibly with art. Neanderthals also used the Levalloisian toolmaking technique, which entails first gradually forming a stone “core” that does not resemble the desired tools and then shifting to an entirely different “pressure-flaking” technique [25]. The Levalloisian technique is conceptually similar to making a cake—first mixing stirring ingredients and then shifting to baking in an oven [26]. These capabilities could not have been transmitted absent language.

Art generally is accepted as a mark for human language. The archaeological record points to art being present in humans before the date for the appearance of language proposed by Bolhuis et al. [2]. The complex paint-making process carried out at the Blombos cave site in Africa, which involved simmering and stirring pigments and binders for extended periods, occurred 100,000 years ago [27]. This complex, labor-intensive enterprise would not have existed without earlier, simpler uses of red ochre and other pigments for ornamentation and painting [7]. Moreover, the presence or absence of art doesn’t tell us anything about the syntactic structure of a language. The Piraha people possess art, but their sentences, lacking embedded clauses [28], don’t conform to the minimalist definition of language proposed by Bolhuis et al. [2].
